# Retrospective Cohort Study on the Association Between a Laboratory Test‐Derived Frailty Index and Mortality in Elderly Critically Ill Patients With Chronic Kidney Disease

**DOI:** 10.1002/hsr2.73331

**Published:** 2026-10-08

**Authors:** Song Zhou, Yi Yu

**Affiliations:** ^1^ Department of Anesthesiology, Qingyuan People's Hospital The Sixth Affiliated Hospital of Guangzhou Medical University Qingyuan Guangdong Province People's Republic of China; ^2^ Department of Critical Care Medicine The Second Affiliated Hospital of Guangzhou University of Chinese Medicine Guangzhou Guangdong People's Republic of China

**Keywords:** chronic kidney disease, FI‐Lab, ICU, MIMIC‐IV, mortality

## Abstract

**Background/Aims:**

Chronic kidney disease (CKD) represents a growing worldwide health concern. There is a need for easily obtainable markers that can improve risk evaluation in affected patients. Here, we investigated whether a laboratory‐derived frailty index (FI‐Lab) carries prognostic information for older CKD individuals admitted to intensive care.

**Methods:**

A retrospective cohort design was adopted, drawing on the MIMIC‐IV repository. Records of elderly ICU patients with diagnosed CKD were retrieved. The endpoints of interest were all‐cause death within 30 and 90 days. Cox proportional hazards regression with incremental covariate adjustment and propensity score matching were employed to quantify the independent contribution of FI‐Lab to survival.

**Results:**

The analytic sample consisted of 5013 hospitalized CKD patients (mean age 77.9 ± 7.6 years; 61.4% male). Following full adjustment, a 0.1‐unit rise in FI‐Lab corresponded to a 94% higher mortality risk at 30 days (HR 1.94; 95% CI 1.57–2.40) and a 65% higher risk at 90 days (HR 1.65; 95% CI 1.43–1.91; both *p* < 0.001). Propensity‐matched analyses yielded comparable estimates. No meaningful association emerged between FI‐Lab and ICU or hospital length of stay. Conversely, higher FI‐Lab values were accompanied by greater odds of acute kidney injury and continuous renal replacement therapy utilization.

**Conclusions:**

An elevated FI‐Lab score at ICU entry is independently tied to elevated short‐ and intermediate‐term mortality in older critically ill patients with CKD. Confirmatory prospective work is needed to establish its clinical role in this population.

## 1 Introduction

An estimated 11%–16% of adults globally live with CKD, a condition that carries substantial economic and healthcare costs [1, 2]. Mortality rates among CKD patients and those on dialysis far exceed those of individuals without kidney disease [3, 4]. Frailty is especially common in this group, with prevalence climbing further in older age [5]. Older adults often contend with hypertension, diabetes, cardiovascular and cerebrovascular disorders, and other chronic illnesses that require sustained pharmacologic and surgical management, raising the likelihood of developing CKD [6, 7, 8, 9]. Frailty exerts a major influence on therapeutic outcomes, longevity, daily functioning, and quality of life in geriatric patients; consequently, its recognition is essential for thorough clinical assessment.

Frailty describes a geriatric condition defined by depleted physiological reserves and diminished resilience against acute insults such as infection or trauma [10]. While occasionally mistaken for disability or multimorbidity, it is currently regarded as a separate clinical construct carrying its own prognostic weight [11]. Operating along a continuum akin to biological age, frailty forecasts unfavorable outcomes irrespective of chronological age, sex, or concurrent diseases [12]. Within the ICU setting, evaluating frailty demands familiarity with the underlying pathological mechanisms active in critically ill patients [13]. In this environment, frailty manifests as a composite picture of advancing age, accumulated comorbidity, nutritional compromise, and multi‐system deterioration, together signaling a constrained capacity to endure physiological challenges [10, 14]. Bedside frailty detection is complicated by the fact that standard tools rely on direct patient contact and devices such as grip strength meters [15]. To circumvent this obstacle, Howlett and colleagues proposed the FI‐Lab, an objective frailty metric derived from routine laboratory tests and vital sign recordings [16]. This index has exhibited robust discriminatory power and outcome prediction capability in varied clinical cohorts [17, 18, 19, 20, 21]. Therefore, it holds promise as a practical frailty screening option for ICU patients with CKD.

Using the MIMIC‐IV repository, we derived FI‐Lab values from admission‐day clinical data of older ICU patients spanning 2008–2022 and examined their relationship with survival and other endpoints. We anticipated that elevated FI‐Lab would track with higher death rates and extended ICU and hospital stays. The results may aid risk stratification and guide care decisions in this susceptible group.

## 2 Methods

### 2.1 Data Source and Patient Population

Patient‐level information was extracted from the MIMIC‐IV resource (version 3.0), a de‐identified critical care registry encompassing admissions to Beth Israel Deaconess Medical Center in Boston, Massachusetts, spanning 2008 through 2022 [22]. Data access was granted to Y.Y. under a PhysioNet credentialed user agreement (certificate identifier: 6477678). Eligible participants were those aged 65 years or older with a recorded CKD diagnosis who experienced a first‐time ICU admission. CKD identification relied on ICD‐10 N18.x codes appearing in discharge summaries or historical medical records. Cases where the earliest renal impairment code surfaced after ICU Day 3 were removed to reduce the chance of confusing acute kidney injury with preexisting CKD. Further grounds for exclusion comprised age below 65 years, ICU residence under 24 h, and lack of more than six of the 33 FI‐Lab constituent variables. The study was conducted according to the STROBE reporting guidelines [23].

### 2.2 Ethics Approval and Consent to Participate

The original data collection for MIMIC‐IV was approved by the Institutional Review Boards of the Massachusetts Institute of Technology and Beth Israel Deaconess Medical Center (approval number: 0403000206). Access to the database was granted to the author (Yi Yu) after completion of the Collaborative Institutional Training Initiative (CITI) program for human research protections (certification number: 6477678). Written informed consent was obtained from participants or their legal representatives at the time of enrollment. As the present study involved secondary analysis of de‐identified, publicly available data, it was exempt from additional IRB approval.

### 2.3 Data Extraction and Variables

Retrieved data covered demographic details, vital sign measurements, laboratory results, comorbid conditions, illness severity indices (SAPS II, SOFA), and ICU‐level interventions. The FI‐Lab was assembled from 34 candidate items encompassing hematologic and biochemical panels, urinalysis, arterial blood gas parameters, and three vital signs, each averaged across the initial 24‐h ICU window. Every item was dichotomized according to the institutional reference intervals embedded in the MIMIC‐IV schema: a reading outside the normal boundary received a score of 1, while a reading within range was coded as 0. The aggregate FI‐Lab value was computed as the fraction of abnormal items relative to the total number of non‐missing entries, yielding a continuous metric bounded by 0 and 1. Supporting Information S1: Table S1 enumerates all constituent variables together with their respective threshold definitions.

### 2.4 Covariates and Multicollinearity Assessments

Covariates included age, sex, race, body mass index (BMI), hospital length of stay, 30‐ and 90‐day vital status, comorbidities (congestive heart failure, myocardial infarction, diabetes, cerebrovascular disease, renal disease, malignancy, sepsis, and severe liver disease), and ICU interventions (vasoactive drug use, mechanical ventilation, and continuous renal replacement therapy [CRRT]). Multicollinearity was evaluated using the variance inflation factor (VIF), with a cutoff of VIF > 2 considered indicative of concerning collinearity.

### 2.5 Outcome Measures

Primary outcomes were all‐cause mortality at 30 and 90 days post‐ICU admission. Secondary outcomes included ICU and hospital length of stay, incidence of acute kidney injury (AKI), and receipt of CRRT.

### 2.6 Statistical Analysis

#### 2.6.1 Baseline Characteristic Assessment

Continuous variables were reported as means with standard deviations or medians with interquartile ranges, with group comparisons conducted via analysis of variance or Kruskal–Wallis testing as dictated by distributional properties. Categorical data were presented as frequencies and percentages and contrasted using *χ* ^2^ or Fisher's exact tests.

Across all covariates, the proportion of missingness fell below 1%; missing continuous entries were replaced by the median. The shape of the FI‐Lab–mortality relationship was examined with restricted cubic splines featuring three knots positioned at the 10th, 50th, and 90th percentiles. Multivariable Cox proportional hazards modeling was carried out to gauge the independent mortality association of FI‐Lab. Four hierarchical models were built, each incorporating successive layers of covariate control. Subgroup evaluations and interaction terms were employed to detect possible effect modifiers. For added rigor, 1:1 propensity score matching was executed using a nearest‐neighbor algorithm with a caliper of 0.1, achieving balance on baseline attributes between surviving and deceased patients.

#### 2.6.2 Survival Analysis

Survival probabilities were estimated using the Kaplan–Meier method, with comparisons across FI‐Lab tertiles performed via the log‐rank test. Stratified analyses and interaction tests were conducted for the following prespecified subgroups: age (< 75 vs. ≥ 75 years), BMI (< 25 vs. ≥ 25 kg/m^2^), sex (male vs. female), race (White vs. other), sepsis (present vs. absent), diabetes (none, without complications, with complications), hypertension (yes vs. no), and congestive heart failure (yes vs. no).

#### 2.6.3 Software and Statistical Methods

All analyses were conducted using STATA (version 17.0), supplemented by R software (http://www.R-project.org; R Foundation for Statistical Computing) and Free Statistics software (version 1.8) [24]. A two‐tailed *p* value < 0.05 was considered statistically significant.

## 3 Results

### 3.1 Characteristics of Patients and Baseline Conditions

The patient selection pathway is illustrated in Figure 1. From an initial pool of 13,746 individuals with CKD satisfying broad entry criteria, 5013 were retained after removing cases with repeated ICU stays, age younger than 65 years, ICU occupancy under 24 h, or excessive missing FI‐Lab elements. The ultimate cohort averaged 77.9 ± 7.6 years of age, and 61.4% were male.

**Figure 1:**
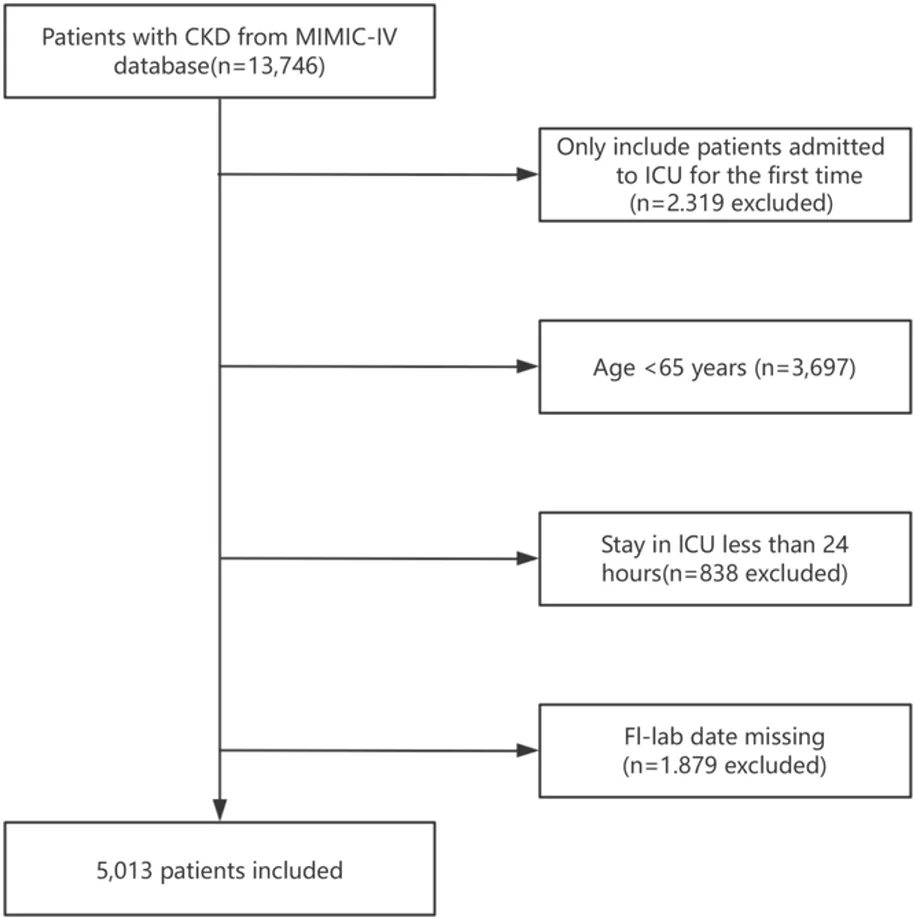
Flow of patient enrollment.

Table 1 displays baseline profiles grouped by FI‐Lab category. Relative to those in the high FI‐Lab tier, participants in the low FI‐Lab stratum exhibited less frequent sepsis, vasopressor support, mechanical ventilation, and CRRT administration, together with lower SAPS II and SOFA scores.

**Table 1:** Baseline characteristics of participants.

Variables	Total (*n* = 5013)	Low Fi‐lab (*n* = 2799)	High Fi‐lab (*n* = 2214)	*p*
Age, years	77.9 ± 7.6	78.0 ± 7.7	77.8 ± 7.6	0.208
Sex, male, *n* (%)	3078 (61.4)	1692 (60.5)	1386 (62.6)	0.12
BMI, kg/m^2^	28.5 ± 6.7	28.2 ± 6.3	28.9 ± 7.1	0.009
Race, *n* (%)				0.064
White	3117 (62.2)	1772 (63.3)	1345 (60.7)	
Others	1896 (37.8)	1027 (36.7)	869 (39.3)	
Marital status				0.409
Married, *n* (%)	2365 (47.2)	1335 (47.7)	1030 (46.5)	
Unmarried, *n* (%)	2648 (52.8)	1464 (52.3)	1184 (53.5)	
Insurance, *n* (%)				0.498
Medicaid	225 (4.5)	126 (4.5)	99 (4.5)	
Medicare	4393 (87.6)	2467 (88.1)	1926 (87)	
Private	325 (6.5)	169 (6)	156 (7)	
Others	70 (1.4)	37 (1.3)	33 (1.5)	
Saps‐ii	46.6 ± 13.5	42.7 ± 11.2	51.6 ± 14.4	< 0.001
SOFA score	6.8 ± 3.7	5.7 ± 3.1	8.3 ± 3.9	< 0.001
Charlson comorbidity index	9.0 ± 2.1	8.9 ± 2.0	9.3 ± 2.1	< 0.001
Hypertension, *n* (%)	851 (17.0)	510 (18.2)	341 (15.4)	0.008
Sepsis, *n* (%)	3430 (68.4)	1693 (60.5)	1737 (78.5)	< 0.001
MI, *n* (%)	1413 (28.2)	775 (27.7)	638 (28.8)	0.378
CHF, *n* (%)	2805 (56.0)	1500 (53.6)	1305 (58.9)	< 0.001
CBVD, *n* (%)	792 (15.8)	498 (17.8)	294 (13.3)	< 0.001
CPD, *n* (%)	1442 (28.8)	796 (28.4)	646 (29.2)	0.566
Diabetes, *n* (%)				< 0.001
Non‐diabetes	2545 (50.8)	1490 (53.2)	1055 (47.7)	
Without complications	866 (17.3)	478 (17.1)	388 (17.5)	
With complications	1602 (32.0)	831 (29.7)	771 (34.8)	
Malignant cancer, *n* (%)	659 (13.1)	303 (10.8)	356 (16.1)	< 0.001
Severe liver disease, *n* (%)	170 (3.4)	57 (2)	113 (5.1)	< 0.001
CRRT, *n* (%)	457 (9.1)	138 (4.9)	319 (14.4)	< 0.001
Vasoactive drug, *n* (%)	2072 (41.3)	939 (33.5)	1133 (51.2)	< 0.001
Ventilation, *n* (%)	2103 (42.0)	1030 (36.8)	1073 (48.5)	< 0.001
30‐Day mortality	202 (4.0)	41 (1.5)	161 (7.3)	< 0.001
90‐Day mortality	378 (7.5)	102 (3.6)	276 (12.5)	< 0.001

*Note:* For each variable, mean ± standard deviation, median (interquartile range), or number (percentage) is reported (as appropriate).

Abbreviations: BMI, body mass index; CBVD, cerebrovascular disease; CHF, congestive heart failure; CPD, chronic pulmonary disease; CRRT, continuous renal replacement therapy, ICU, intensive care unit; MI, myocardial infarct; SAPS II, simplified acute physiology score II; SOFA, sequential organ failure assessment.

### 3.2 The Linear Association Between FI–Lab Scores and Mortality Rate

Restricted cubic spline analysis revealed a graded, monotonic relationship between FI‐Lab at ICU admission and in‐hospital mortality (*p* for nonlinearity = 0.557). As displayed in Figure 2, mortality risk increased progressively with rising FI‐Lab scores.

**Figure 2:**
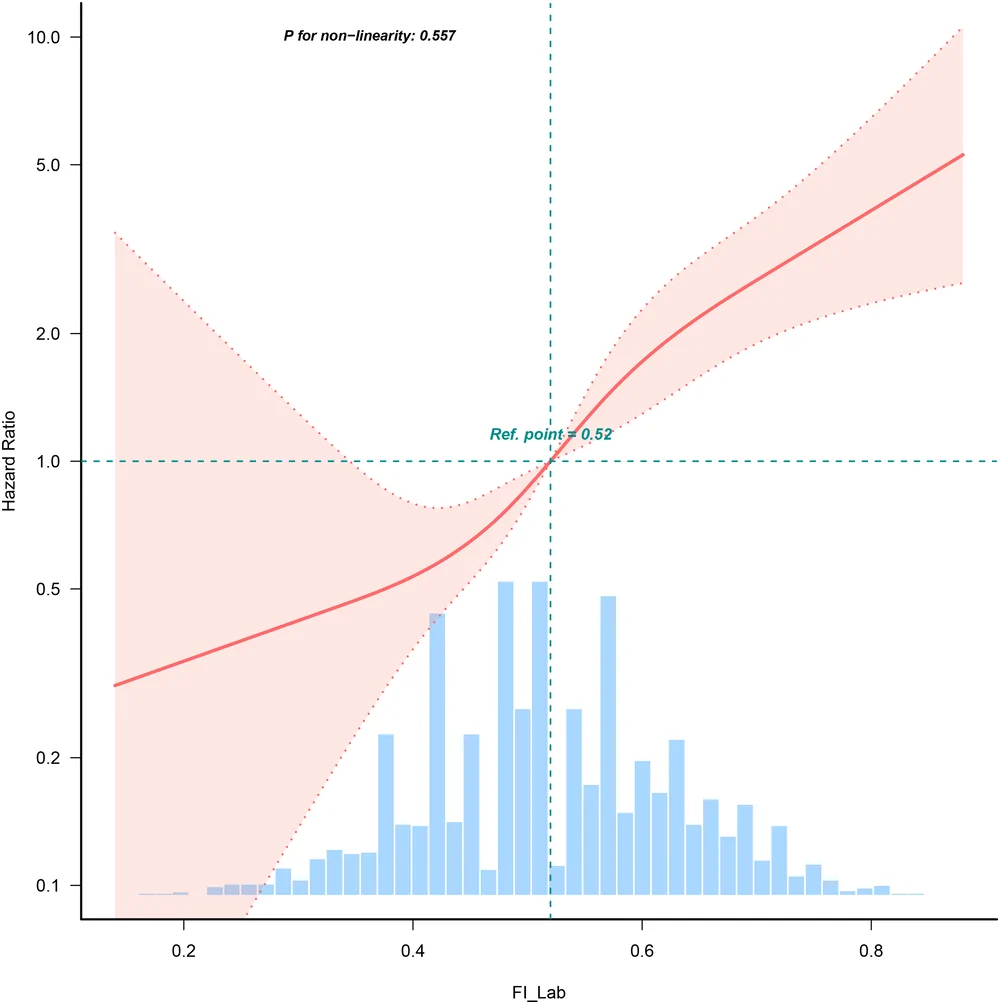
Restricted cubic spline plot depicting the dose‐response relationship between FI‐Lab (continuous) and 90‐day mortality, adjusted for all covariates in a multivariable Cox model 3.

On univariable analysis, FI‐Lab was significantly associated with both 30‐day mortality (HR 2.39; 95% CI 2.11–2.71) and 90‐day mortality (HR 2.02; 95% CI 1.84–2.21; both *p*< 0.001). These associations persisted across all four multivariable Cox models (Table 2). After full covariate adjustment, each 0.1‐unit increase in FI‐Lab conferred a 94% higher hazard of 30‐day mortality (HR 1.94; 95% CI 1.57–2.40) and a 65% higher hazard of 90‐day mortality (HR 1.65; 95% CI 1.43–1.91; both *p* < 0.001). The results remained consistent across all modeling strategies.

**Table 2:** Univariate and multivariate Cox regression models of FI‐Lab with mortality in patients with CKD.

	Unadjusted model	Model 1	Model 2	Model 3	PSM
Variable	HR 95% CI	HR 95% CI	HR 95% CI	HR 95% CI	HR 95% CI
30‐Day mortality					
FI‐lab (per 0.1 score)	2.39 (2.11–2.71)	3 (2.47–3.64)	2.32 (1.88–2.88)	1.94 (1.57–2.4)	1.88 (1.46–2.41)
FI‐lab (as a categorical variable)					
< 0.53	1 (Ref)	1 (Ref)	1 (Ref)	1 (Ref)	1 (Ref)
≥ 0.53	5.12 (3.64–7.22)	7.76 (4.32–13.93)	4.89 (2.64–9.05)	5 (2.64–9.46)	4.01 (2.08–7.75)
90‐Day mortality					
FI‐lab (per 0.1 score)	2.02 (1.84–2.21)	2.36 (2.07–2.69)	1.86 (1.61–2.16)	1.65 (1.43–1.91)	1.68 (1.41–2)
FI‐lab (as a categorical variable)					
< 0.53	1 (Ref)	1 (Ref)	1 (Ref)	1 (Ref)	1 (Ref)
≥ 0.53	3.61 (2.88–4.53)	4.76 (3.37–6.72)	2.99 (2.07–4.32)	3.02 (2.06–4.43)	2.94 (1.94–4.47)

Abbreviations: CI, confidence interval; CKD, chronic kidney disease; FI‐Lab, physiological and laboratory‐based frailty index; HR, hazard ratio; Ref, reference; PSM, propensity score matching.

Model 1: Adjusted for age, sex, and BMI.

Model 2: Adjusted for Model 1, race, insurance, marital status, SAPS II, SOFA, and Charlson comorbidity index.

Model 3: Adjusted for Model 2, hypertension, sepsis, myocardial infarct, congestive heart failure, cerebrovascular disease, chronic pulmonary disease, diabetes, malignant cancer, severe liver disease, CRRT, ventilation, and vasoactive drugs.

PSM: Based on Model 3.

### 3.3 The Analysis of the Kaplan–Meier Survival Curves

Kaplan–Meier curves (Figure 3) demonstrated that patients with higher FI‐Lab scores had significantly shorter cumulative survival over the follow‐up period (log‐rank *p* < 0.001).

**Figure 3:**
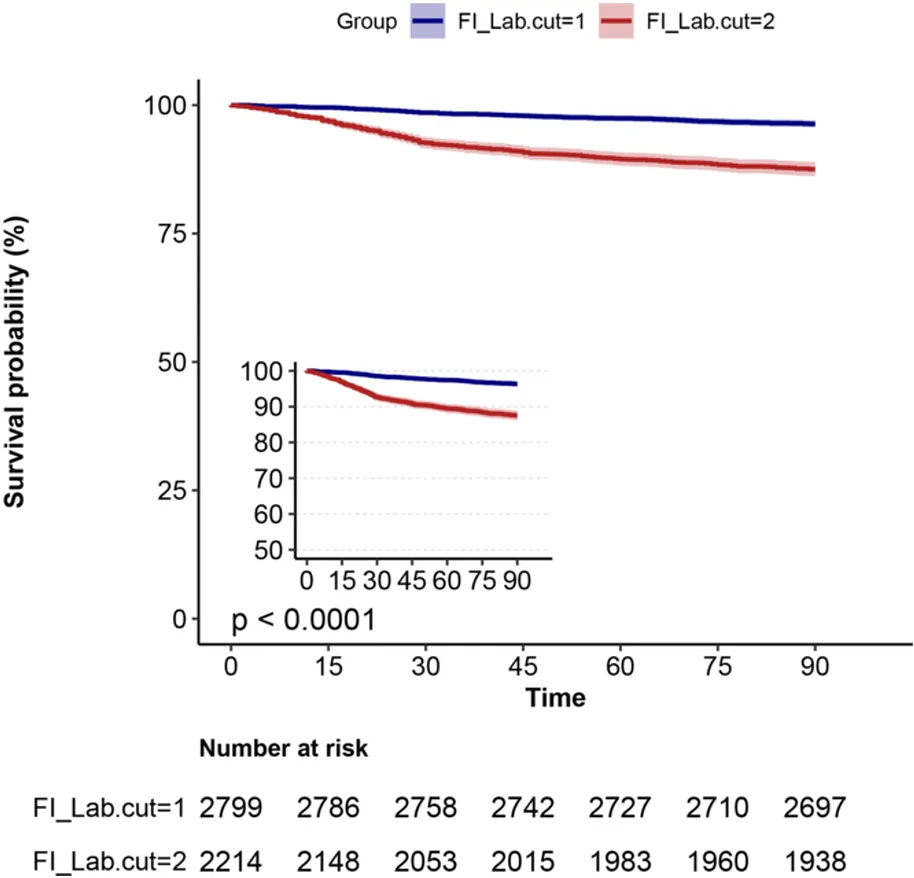
Kaplan–Meier survival estimates at 90 days stratified by FI‐Lab tertile among CKD patients.

### 3.4 Relationship Between FI‐Lab Scores and Other Outcomes

After full adjustment (Table 3), FI‐Lab showed no significant association with ICU length of stay (*β* = −0.18; 95% CI −0.43 to 0.07) or hospital length of stay (*β* = −0.24; 95% CI −0.67 to 0.19). Similar null findings were obtained when FI‐Lab was modeled as a dichotomous variable (*β* = 0.08; 95% CI −0.58 to 0.73 for ICU stay; *β* = 0.11; 95% CI −0.83 to 1.05 for hospital stay).

**Table 3:** Associations of the FI‐Lab with ICU stay and hospital stay among CKD.

	Length of stay in the ICU (days)			Length of stay in the hospital (days)		
	Model 1	Model 2	PSM	Model 1	Model 2	PSM
Variable	*β* (95% CI)	□*β* (95% CI)	□*β* (95% CI)	□*β* (95% CI)	□*β* (95% CI)	□*β* (95% CI)
FI‐lab (per 0.1 score)	0.41 (0.28~0.55)	−0.18 (−0.43~0.07)	−0.21 (−0.53~0.1)	0.71 (0.46~0.97)	−0.24 (−0.67~0.19)	−0.3 (−0.84~0.24)
FI‐lab (as a categorical variable)						
< 0.53	0 (Ref)	0 (Ref)	0 (Ref)	0 (Ref)	0 (Ref)	0 (Ref)
≥ 0.53	0.95 (0.63~1.27)	0.04 (−0.51~0.59)	0.08 (−0.58~0.73)	1.69 (1.08~2.3)	0.11 (−0.83~1.05)	0.04 (−1.08~1.17)

Abbreviations: CI, confidence interval; CKD, chronic kidney disease; FI‐Lab, physiological and laboratory‐based frailty index; ICU, intensive care unit; Ref, reference; PSM, propensity score matching.

Model 1: Unadjusted.

Model 2: Adjusted for age, sex, BMI, race, insurance, marital status, SAPS II, SOFA, Charlson comorbidity index, hypertension, sepsis, myocardial infarct, congestive heart failure, cerebrovascular disease, chronic pulmonary disease, diabetes, malignant cancer, severe liver disease, CRRT, ventilation, and vasoactive drug.

PSM: Based on Model 2.

Higher FI‐Lab scores were associated with an elevated risk of AKI by ICU Day 7 and a greater likelihood of CRRT receipt. Each 0.1‐unit increase in FI‐Lab corresponded to a 3% higher odds of AKI (OR 1.03; 95% CI 0.93–1.14) and a 28% higher odds of CRRT (OR 1.28; 95% CI 1.13–1.46). When FI‐Lab was dichotomized, the corresponding ORs were 0.92 (95% CI 0.72–1.16) for AKI and 1.59 (95% CI 1.22–2.09) for CRRT (Table 4).

**Table 4:** Associations of the use of FI‐Lab and AKIs and CRRTs among CKD.

Variable	AKI			CRRT		
	Model 1	Model 2	PSM	Model 1	Model 2	PSM
	OR (95% CI)	OR (95% CI)	OR (95% CI)	OR (95% CI)	OR (95% CI)	OR (95% CI)
FI‐lab (per 0.1 score)	1.53 (1.44~1.62)	1.03 (0.93~1.14)	1.03 (0.9~1.17)	1.82 (1.67~1.99)	1.28 (1.13~1.46)	1.34 (1.15~1.56)
FI‐lab (as a categorical variable)						
< 0.53	1 (Ref)	1 (Ref)	1 (Ref)	1(Ref)	1 (Ref)	1 (Ref)
≥ 0.53	1.99 (1.74~2.28)	0.92 (0.72~1.16)	0.96 (0.73~1.26)	3.14 (2.55~3.85)	1.59 (1.22~2.09)	1.63 (1.2~2.22)

Abbreviations: CI, confidence interval; CKD, chronic kidney disease; FI‐Lab, physiological and laboratory‐based frailty index; ICU, intensive care unit; Ref, reference; PSM, propensity score matching.

Model 1: Unadjusted.

Model 2: Adjusted for age, sex, BMI, race, insurance, marital status, SAPS II, SOFA, Charlson comorbidity index, hypertension, sepsis, myocardial infarct, congestive heart failure, cerebrovascular disease, chronic pulmonary disease, diabetes, malignant cancer, severe liver disease, CRRT, ventilation, and vasoactive drug.

PSM: Based on Model 2.

### 3.5 Subgroup and Sensitivity Analysis Results

Subgroup analyses confirmed a consistent mortality association across all predefined strata, with no significant interactions (*p* for interaction > 0.05; Figure 4). After 1:1 propensity score matching, 872 well‐balanced pairs were generated (Supporting Information S1: Table S2). In the matched cohort, the HRs for 30‐ and 90‐day mortality were 1.88 (95% CI 1.46–2.41) and 1.68 (95% CI 1.41–2.00), respectively (both *p* < 0.001).

**Figure 4:**
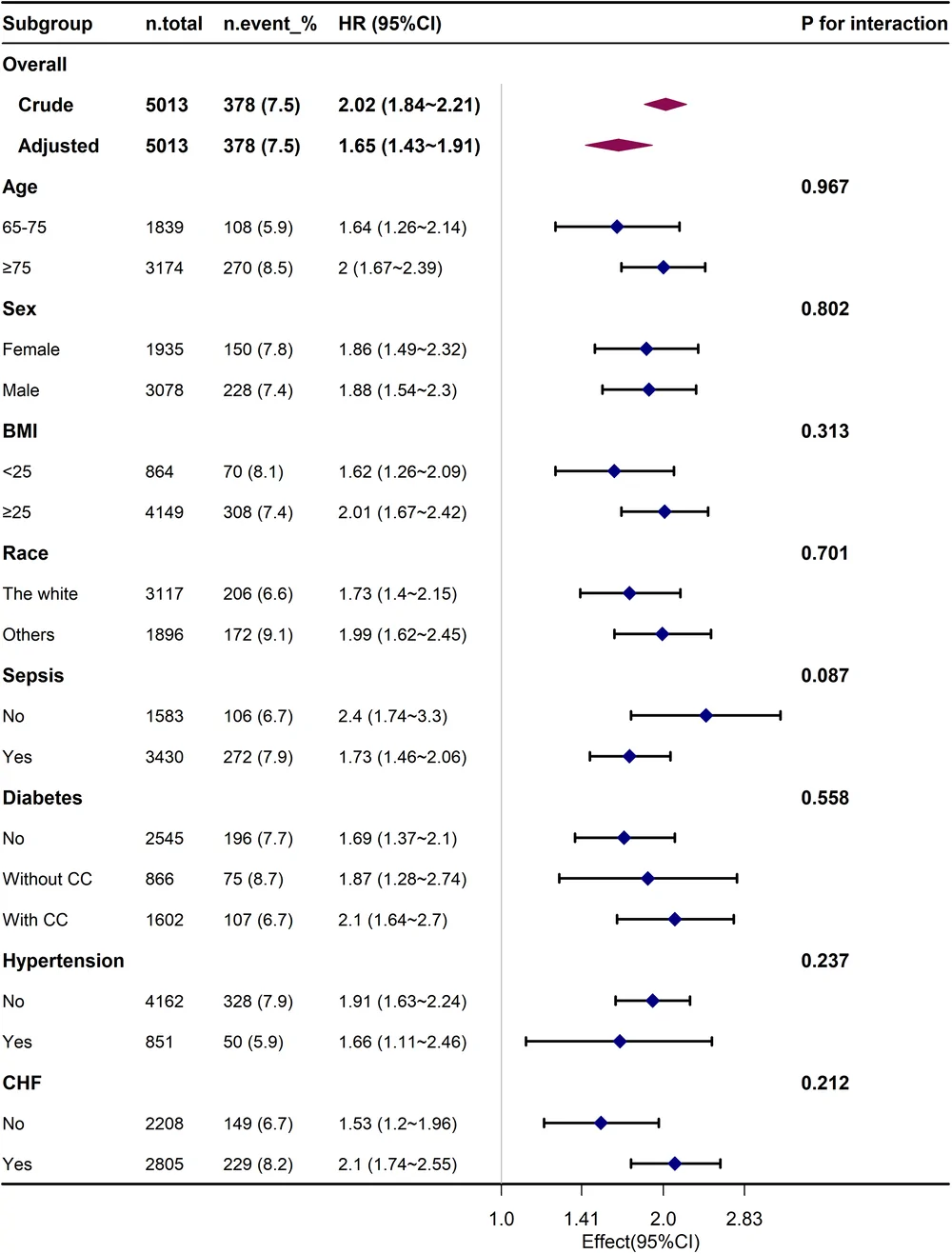
Forest plot summarizing the association between FI‐Lab and 90‐day mortality across predefined subgroups defined by baseline characteristics.

When FI‐Lab was treated as a categorical variable, the association with mortality remained robust, with HRs for 30‐day mortality ranging from 4.89 to 7.76 (all *p*< 0.001). A similar pattern was observed for 90‐day mortality (Table 2). No significant associations were found between categorical FI‐Lab and ICU or hospital length of stay (Table 3), nor between categorical FI‐Lab and AKI or CRRT risk (Table 4).

## 4 Discussion

### 4.1 Key Findings

In this retrospective investigation leveraging the MIMIC‐IV database, we assessed the connection between a laboratory‐based frailty index and short‐ as well as intermediate‐term survival among older ICU patients with CKD. The data show that individuals registering higher FI‐Lab values face substantially greater mortality at both 30 and 90 days. This pattern endured after multivariable adjustment, propensity score matching, and treatment of FI‐Lab as a categorical variable, and was corroborated by subgroup exploration and Kaplan–Meier curve inspection. Restricted cubic spline modeling additionally uncovered a monotonic dose–response gradient between FI‐Lab and death risk. Taken together, these observations suggest that FI‐Lab may serve as a useful adjunct for mortality risk classification in this specific patient group.

Our results indicate that a frailty metric built entirely from routine labs and vitals can independently forecast 30‐ and 90‐day survival in older ICU‐admitted CKD patients. To our knowledge, no prior study has specifically applied FI‐Lab to a CKD‐ICU population with this length of follow‐up. Since the index demands no extra equipment, examiner training, or patient effort, it may serve as a feasible substitute for full geriatric evaluation in ICUs with limited resources. Moreover, FI‐Lab contributed prognostic information beyond what SOFA and SAPS II alone provided, implying that physiological frailty and acute illness severity capture overlapping yet distinct aspects of risk.

Of note, FI‐Lab was not significantly associated with ICU or hospital length of stay. Higher FI‐Lab scores were linked to an increased likelihood of CRRT requirement, but not to AKI risk.

### 4.2 Influence of the FI–Lab on Mortality Among Elderly Critically Ill Patients With CKD

The FI‐Lab has shown consistent prognostic ability in a variety of clinical environments [17, 25, 26]. Emerging work suggests that it can forecast in‐hospital death in mixed ICU populations and, when paired with alternative frailty instruments, may sharpen the detection of high‐risk cases [25]. Nonetheless, evidence regarding its use specifically in older adults with CKD remains limited. Prior studies have linked frailty to roughly a 2.5‐fold rise in the risk of CKD advancement and death [27, 28], along with greater hospitalization rates both in the general population and among those with end‐stage renal disease [4, 27, 29].

The elevated frailty burden in CKD likely arises from disease‐driven mechanisms including protein‐energy depletion, anemia, systemic inflammatory activation, acid‐base disturbance, and hormonal dysregulation [30, 31]. CKD is itself considered a condition of premature metabolic senescence, marked by persistent inflammation, glycation end‐product buildup, oxidative damage, insulin insensitivity, vascular stiffening, and skeletal mineral abnormalities [32, 33]. Many of these alterations mirror the classic frailty phenotype observed in non‐CKD older adults—reduced activity levels, muscle loss, multiple comorbidities, and cognitive impairment [34]. Given that these biologic disruptions are themselves frailty correlates [16, 35], the inverse relationship between glomerular filtration rate and frailty frequency in the general population is expected [36].

In the current analysis, elevated FI‐Lab emerged as a significant predictor of mortality in elderly CKD patients, with risk increasing in parallel with FI‐Lab scores. This relationship persisted after extensive covariate adjustment and PSM. Importantly, frailty is not a fixed state but a potentially modifiable condition; thus, identifying frail patients in the ICU may enable targeted interventions such as early mobilization and nutritional support, which could improve outcomes [37, 38]. Two recent investigations have underscored the interplay among CKD, frailty, and prognosis in older heart failure patients [39, 40]. Although our study did not stratify by CKD severity, the consistent performance of FI‐Lab across multiple comorbidity subgroups—including congestive heart failure—suggests that the laboratory frailty signal operates broadly in this population. Future work with adequate sample sizes across eGFR categories is needed to determine whether the FI‐Lab–mortality gradient varies by renal function stage. Consistent with this, Díez‐Villanueva et al. recently showed that in older heart failure patients, frailty outweighed CKD severity as a mortality predictor [40], while Cheong et al. further demonstrated that malnutrition risk—partially captured by FI‐Lab components such as albumin—predicts kidney function decline in community‐dwelling older adults [41]. Together, these findings support the multidimensional nature of risk captured by FI‐Lab in elderly CKD‐ICU patients.

### 4.3 Association Between FI–Lab and Other Results

Neither continuous nor categorical FI‐Lab showed a significant link to ICU or hospital length of stay in our analysis. Still, unaccounted confounding variables may play a role. Frailty could indirectly shape recovery speed and complication handling, which in turn affect how long patients remain hospitalized. For example, immunocompromise or inadequate nutritional intake in frail individuals might slow recuperation and extend admission duration [42, 43, 44].

In contrast, we found a significant positive association between FI‐Lab and CRRT requirement: each 0.1‐unit increment in FI‐Lab corresponded to a 28% higher odds of needing CRRT. However, no significant link was detected between FI‐Lab and AKI risk. This pattern aligns with prior reports suggesting that frailty is a meaningful prognostic factor for renal deterioration in critically ill patients. For example, one study found that frail patients had a higher likelihood of requiring CRRT after cardiac surgery [45]. These observations corroborate our findings and underscore the predictive value of FI‐Lab for CRRT need. At the same time, our null result for AKI contrasts with earlier work showing that frailty significantly increases AKI risk in ICU populations [46, 47]. This discrepancy may reflect differences in cohort composition; our sample was restricted to older ICU patients, whereas previous studies included broader age ranges. Future investigations incorporating larger samples and additional covariates are warranted to clarify these relationships.

### 4.4 Strengths of This Study

Several methodological strengths deserve acknowledgment. First, reliance on a sizable, openly accessible database bolsters data dependability and external applicability. Second, to our awareness, this constitutes the initial inquiry devoted specifically to the FI‐Lab–mortality relationship in elderly ICU patients with CKD, thereby extending prior literature by spotlighting a novel prognostic indicator for this susceptible population. Third, we deployed stringent analytical techniques, encompassing multivariable regression and propensity score matching, to fortify result validity and curtail confounding. Fourth, because FI‐Lab draws solely on laboratory tests and vital signs already gathered during routine care, it is computationally simple and fits naturally into existing clinical routines. Adoption of this index could equip clinicians to recognize high‐risk CKD patients at an earlier juncture, enabling prompt intervention and customized care planning—such as intensified surveillance and augmented supportive measures for those exhibiting elevated FI‐Lab values.

### 4.5 Limitations

A number of limitations warrant mention. First, FI‐Lab was determined solely at the point of ICU entry; values may shift as the clinical trajectory unfolds. While repeated measurements could illuminate temporal dynamics, the link between evolving FI‐Lab patterns and CKD prognosis in older persons has not been studied. Second, the investigation drew on data from a single U.S.‐based ICU and confined enrollment to older adults with CKD, restricting transportability to alternate settings or patient groups. Third, our database did not contain information on several elements recognized to affect CKD‐associated mortality, such as adequacy of antimicrobial therapy, fluid resuscitation practices, anemia management, blood pressure regulation, and cardiovascular comorbidity treatment. Subsequent studies ought to incorporate these dimensions. Fourth, individuals who underwent CRRT or continuous venovenous hemofiltration during their ICU course were kept in the analytic sample, with CRRT entered as a covariate; consequently, the predictive capacity of FI‐Lab applies to CKD‐ICU patients receiving concurrent renal replacement therapy. However, FI‐Lab captures admission‐day laboratory frailty and should not be viewed as a replacement for dialysis‐burden quantification. Fifth, although the Nutritional Risk Screening 2002 instrument exists within MIMIC‐IV, its extensive missingness in our cohort prevented dependable adjustment for nutritional risk. Certain FI‐Lab constituents, notably albumin and cholesterol, partly reflect nutritional condition. Standardized physical rehabilitation metrics were absent and thus could not be accommodated. Lastly, the retrospective architecture leaves room for residual confounding. Despite controlling for numerous covariates and applying PSM along with doubly robust estimation, unmeasured confounders may still have shaped the observed associations.

## 5 Conclusions

A heightened FI‐Lab value at the time of ICU admission is independently tied to greater 30‐ and 90‐day mortality among older critically ill patients with CKD. Functioning as a frailty surrogate built from ordinary clinical data, FI‐Lab represents an inexpensive, easily accessible risk marker that may supplement established severity scoring systems in this at‐risk group.

## Author Contributions

S.Z. carried out the statistical analyses and prepared the initial draft. S.Z. and Y.Y. jointly performed data analysis and contributed to manuscript revision. Y.Y. handled data acquisition and interpretation. S.Z. and Y.Y. jointly conceptualized the study design and reviewed the final manuscript. All authors have read and given approval to the submitted version.

## Funding

The authors have nothing to report.

## Ethics Statement

The original data collection for MIMIC‐IV was approved by the Institutional Review Boards of the Massachusetts Institute of Technology and Beth Israel Deaconess Medical Center (approval number: 0403000206). Access to the database was granted to the author (Yi Yu) after completion of the Collaborative Institutional Training Initiative (CITI) program for human research protections (certification number: 6477678). Written informed consent was obtained from participants or their legal representatives at the time of enrollment. As the present study involved secondary analysis of de‐identified, publicly available data, it was exempt from additional IRB approval.

## Conflicts of Interest

The authors declare no conflicts of interest.

## Transparency Statement

The authors confirm that this report presents an honest, precise, and open account of the investigation; that no essential elements have been left out; and that any departures from the originally planned protocol (and, where relevant, trial registration) have been disclosed.

## Supporting information


Supporting File 1

Supporting File 2

## Data Availability

The data supporting the findings of this study are available from the MIMIC‐IV database (version 3.0), hosted on PhysioNet at https://physionet.org/content/mimiciv/3.0/ (DOI: 10.13026/6mm1‐ek67). Access restrictions apply; the data were used under a licensed agreement for this study. Data may be obtained from the corresponding author upon reasonable request and with permission from PhysioNet.
