# Supplementary material for: Retrospective Cohort Study on the Association Between a Laboratory Test‐Derived Frailty Index and Mortality in Elderly Critically Ill Patients With Chronic Kidney Disease
Source: Health Sci Rep. 2026 Oct 8;9(10):e73331. doi: 10.1002/hsr2.73331 (PMC13650525; doi:10.1002/hsr2.73331)
Supplement: Supplementary file 2 [file HSR2-9-e73331-s002.docx]

**Table S2:** Baseline characteristics of participants after PSM.

| **Variables** | **Total**  **(n = 1,744)** | **Low Fi-lab**  **(n = 872)** | **High Fi-lab**  **(n = 872)** | **P** |
| --- | --- | --- | --- | --- |
| **Age, y** | 77.3 ± 7.4 | 77.3 ± 7.4 | 77.4 ± 7.4 | 0.867 |
| **Sex, Male, n (%)** | 1121 (64.3) | 561 (64.3) | 560 (64.2) | 0.96 |
| **BMI, kg/m^2^** | 28.4 ± 6.7 | 28.3 ± 6.5 | 28.5 ± 6.8 | 0.657 |
| **Race, n (%)** |  |  |  | 0.693 |
| White | 1088 (62.4) | 540 (61.9) | 548 (62.8) |  |
| Others | 656 (37.6) | 332 (38.1) | 324 (37.2) |  |
| **Marital status,** |  |  |  | 0.848 |
| Married, n (%) | 848 (48.6) | 422 (48.4) | 426 (48.9) |  |
| Unmarried, n (%) | 896 (51.4) | 450 (51.6) | 446 (51.1) |  |
| **Insurance, n (%)** |  |  |  | 0.912 |
| Medicaid | 65 (3.7) | 34 (3.9) | 31 (3.6) |  |
| Medicare | 1538 (88.2) | 771 (88.4) | 767 (88) |  |
| Private | 117 ( 6.7) | 56 (6.4) | 61 (7) |  |
| Others | 24 ( 1.4) | 11 (1.3) | 13 (1.5) |  |
| **Saps-ii** | 47.7 ± 11.7 | 47.9 ± 11.9 | 47.6 ± 11.6 | 0.641 |
| **SOFA score** | 7.7 ± 3.4 | 7.7 ± 3.3 | 7.7 ± 3.5 | 0.848 |
| **Charlson comorbidity index** | 9.0 ± 2.0 | 9.0 ± 2.0 | 9.1 ± 2.0 | 0.3 |
| **Hypertension, n (%)** | 279 (16.0) | 140 (16.1) | 139 (15.9) | 0.948 |
| **Sepsis, n (%)** | 1309 (75.1) | 648 (74.3) | 661 (75.8) | 0.472 |
| **MI, n (%)** | 541 (31.0) | 270 (31) | 271 (31.1) | 0.959 |
| **CHF, n (%)** | 1011 (58.0) | 505 (57.9) | 506 (58) | 0.961 |
| **CBVD, n (%)** | 283 (16.2) | 139 (15.9) | 144 (16.5) | 0.745 |
| **CPD, n (%)** | 492 (28.2) | 249 (28.6) | 243 (27.9) | 0.75 |
| **Diabetes, n (%)** |  |  |  | 0.82 |
| Non-diabetes | 861 (49.4) | 437 (50.1) | 424 (48.6) |  |
| Without complications | 314 (18.0) | 154 (17.7) | 160 (18.3) |  |
| With complications | 569 (32.6) | 281 (32.2) | 288 (33) |  |
| **Malignant cancer, n (%)** | 204 (11.7) | 101 (11.6) | 103 (11.8) | 0.882 |
| **Severe liver disease, n (%)** | 55 (3.2) | 25 (2.9) | 30 (3.4) | 0.493 |
| **CRRT, n (%)** | 229 (13.1) | 91 (10.4) | 138 (15.8) | < 0.001 |
| **Vasoactive drug, n (%)** | 960 (55.0) | 485 (55.6) | 475 (54.5) | 0.63 |
| **Ventilation, n (%)** | 1104 (63.3) | 590 (67.7) | 514 (58.9) | < 0.001 |
| **30-day mortality** | 58 (3.3) | 12 (1.4) | 46 (5.3) | < 0.001 |
| **90-day mortality** | 118 (6.8) | 32 (3.7) | 86 (9.9) | < 0.001 |

Abbreviations: PSM, Propensity-score matching; BMI, body mass index; SAPS II, simplified acute physiology score II; SOFA, sequential organ failure assessment; MI, myocardial infarct; CHF, congestive heart failure; CBVD, cerebrovascular disease; CPD, chronic pulmonary disease; ICU, intensive care unit; CRRT, continuous renal replacement therapy, CRRT, continuous renal replacement therapy; SOFA, continuous renal replacement therapy, SAPS II, simplified acute physiology score II.
